# Genome-Wide Association Analysis of Age-Dependent Egg Weights in Chickens

**DOI:** 10.3389/fgene.2018.00128

**Published:** 2018-04-26

**Authors:** Zhuang Liu, Congjiao Sun, Yiyuan Yan, Guangqi Li, Guiqin Wu, Aiqiao Liu, Ning Yang

**Affiliations:** ^1^National Engineering Laboratory for Animal Breeding and MOA Key Laboratory of Animal Genetics and Breeding, College of Animal Science and Technology, China Agricultural University, Beijing, China; ^2^Beijing Engineering Research Center of Layer, Beijing, China

**Keywords:** egg weight, genome-wide association study, chicken, candidate genes, heritability

## Abstract

Egg weight (EW) is an economically-important trait and displays a consecutive increase with the hen's age. Because extremely large eggs cause a range of problems in the poultry industry, we performed a genome-wide association study (GWAS) in order to identify genomic variations that are associated with EW. We utilized the Affymetrix 600 K high density SNP array in a population of 1,078 hens at seven time points from day at first egg to 80 weeks age (EW80). Results reveal that a 90 Kb genomic region (169.42 Mb ~ 169.51 Mb) in GGA1 is significantly associated with EW36 and is also potentially associated with egg weight at 28, 56, and 66 week of age. The leading SNP could account for 3.66% of the phenotypic variation, while two promising genes (DLEU7 and MIR15A) can be mapped to this narrow significant region and may affect EW in a pleiotropic manner. In addition, one gene (CECR2 on GGA1) and two genes (MEIS1 and SPRED2 on GGA3), which involved in the processes of embryogenesis and organogenesis, were also considered to be candidates related to first egg weight (FEW) and EW56, respectively. Findings in our study could provide worthy theoretical basis to generate eggs of ideal size based on marker assisted breeding selection.

## Introduction

Egg weight (EW) is an economically-important trait in chicken and one of the major indexes used by consumers when directly selecting suitable products (Sasaki et al., 2004). Improvements in the production performance of laying hens have meant that commercial individual ages have been extended from their original 72 week of age (EW72) to EW80 week of age; some companies have even extended the laying cycle to 100 weeks and proposed the breeding program called “Breeding for 500 eggs in 100 weeks” (Schulte-Drüggelte and Thiele, 2013; Bain et al., 2016). Although EW increases in consecutive increments over the entire laying period as hens age (Tumová and Gous, 2012), extremely large eggs cause a range of problems in the poultry industry (including a rapid decline in quality, an increase in breakage rate, and a decreased commercialization rate), especially in the late laying stage (Koelkebeck et al., 2001), while EW also exerts significant effects on hatchability (Nangsuay et al., 2011). This means that controlling EW is very important for breeders if they are to achieve their programs as well as extend the laying cycle.

It is well-known that EW is a highly heritable quantitative trait (Savegnago et al., 2011; Yi et al., 2014). Thus, in concert with developments in molecular genetics, research has been carried out to elucidate the genetic basis of EW (Schreiweis et al., 2006; Wright et al., 2006; Liu et al., 2011; Wolc et al., 2012, 2014; Yi et al., 2015; Liao et al., 2016); a total of 248 quantitative trait loci (QTLs) on 19 different chromosomes (18 autosomes and one sex chromosome) have been reported to be associated with EW and are listed in the AnimalQTLdatabase (https://www.animalgenome.org/cgi-bin/QTLdb/index). However, despite a range of studies in this area, wide confidence intervals for the positions of QTL remain that have rarely been replicated. A new research era was initiated with advances in single nucleotide polymorphism (SNP) chip and sequencing technology, and genome wide association study (GWAS) has become one of the most effective methods to detect genetic variation in livestock. In previous studies, Liu et al. (2011) and Wolc et al. (2012) performed GWASs using moderate density SNP chips to determine associations with EW in chicken, while Yi et al. (2015) subsequently reported several candidate genes that are related to longitudinal EWs using a 600 K high density SNP chip in a GWAS. The *NCAPG* gene, one of those promising genes, was located on GGA4 and may affect egg weight in a pleiotropic manner. Genetic improvements to EW remain slow, however, because of measurement difficulties and other complicating factors.

A very limited number of previous genetic studies have addressed EW across the whole chicken laying period, from the first egg to 80 weeks of age, and most have concentrated on peak or early stage eggs. We therefore utilize a commercial chicken 600 K SNP chip in this study to detect the genetic variations associated with EW at different ages within a population of 1,078 hens using GWAS.

## Materials and methods

### Ethics statement

All the blood samples used in this study were collected in accordance with the Guidelines for Experimental Animals established by the Ministry of Agricultural of China (Beijing, China). The whole of this study was approved by the Animal Welfare Committee of China Agricultural University (permit number: SYXK 2007-0023).

### Resource population

An 11th generation pure line of Rhode Island Red chicken from Beijing Huadu Yukou Poultry Breeding Co., Ltd. comprised the experimental material for this study. A total of 92 sires and 801 dams contributed to this population which has been selected for egg production and quality over many years. Thus, a total of 1,078 hens of accurate known pedigree were chosen for SNP genotyping in order to collect phenotypic EW-related data. All birds were housed in individual cages of the same area at 13 weeks of age and were provided with free access to feed and water.

### Phenotypic measurements and heritability evaluation

We measured EWs at seven different time points in order to depict genetic architecture across the whole laying period. Thus, first egg weight (FEW) is regarded as the weight of the first egg laid by each bird; however, because of the changes in egg laying performance, we collected fresh eggs at 28 weeks of age, 36 weeks of age, 56 weeks of age, 66 weeks of age, 72 weeks of age, and 80 weeks of age for two successive days during the early laying period (before or at 56 weeks) as well as for three successive days during the late laying period (after 56 week). These time points were selected according to the actual breeding objectives of the company, and average EW for either 2 or 3 days was defined as the phenotypic value for each sample. Descriptive statistics of all phenotypic data were handled using the software R version 3.3.1 (https://www.r-project.org/).

Pedigree-based hereditability values for EWs across the whole laying period were calculated using the average information restricted maximum likelihood (AI-REML) method implemented in the software DMU v6.0 (Madsen and Jensen, 2013). A multi-trait general animal model was utilized in this analysis, as follows:

(1) y=1μ+Za+e

In this expression, y denotes the phenotypic value of traits, **1** is the *n* × 1 vector of all 1's, μ refers to population means (fixed effect), **Z** is the *n* × 1 vector of the covariate (random effect), and **a** and **e** denote the additive effect and random residual, respectively.

### Genotyping, quality control, and imputation

We isolated genomic DNA from whole blood samples using standard phenol/chloroform methods, and genotyped qualified DNA of 1,078 hens with the Affymetrix 600 K chicken SNP chip (Affymetrix, Inc. Santa Clara, CA, USA; Kranis et al., 2013). Two linkage groups and two sex chromosomes, 6,550 SNPs with unknown physical positions, and 43 markers with repeated genomic coordinates were excluded from a preliminary set of 580,961 SNPs across 28 autosomes. We then employed the software Affymetrix Power Tools v1.19.0 (APT) to carry out genotype calling and quality control following the Axiom Genotyping Solution pipeline so that individual dish quality control (DQC) was >0.82 and call rate was >97%. A suite of ps-metrics supplied by Affymetrix (http://affymetrix.com/) were then applied to calculate SNP quality values; lower quality ones were filtered out using a bespoke R script so that 1,063 individuals and 517,856 SNPs remained. In addition, we discarded SNPs on sex chromosomes because the egg weight is a quantitative trait and the genes that affecting egg weight are mainly located on autosomes. It is very difficult to detect associations between phenotypes and genotypes in these cases. The software PLINK (Purcell et al., 2007) was then used for further quality control so that minor allele frequencies (MAF) in this analysis were >0.01 and Hardy Weinberg equilibrium (HWE) *P*-values were < 1e-6. The remaining SNPs were utilized to impute some missing genotypes using the software Beagle v4.0 (Browning and Browning, 2009). A final total of 294,705 SNPs and 1,063 individuals were deemed eligible for subsequent genome-wide analyses.

### Population structure and association analysis

We initially performed a principal component analysis (PCA) using the software PLINK to evaluate population stratification prior to GWAS. Considering that clusters of highly correlated SNPs may distort the resulting PCs, all SNPs were pruned to ensure their independence using the indep-pair-wise option in PLINK software, with a window size of 25 SNPs, a step of five SNPs and a r2 threshold of 0.2. And the top five principal components calculated by those independent SNPs were treated as covariates and included in model of GWAS as fixed effects to control for population structure. Considering the possibility of over-conservation inherent to the Bonferroni method, we adjusted the threshold of genome-wide significant *P*-values using the software simpleM (Gao et al., 2010). A total of 31,589 independent effective tests were therefore obtained, and genome-wide and suggestive significance values were calculated as 1.58e-6 (0.05/31,589) and 3.17e-5 (1.00/31,589), respectively.

We initially performed a univariate GWAS by applying a linear mixed model to account for associations between EWs and effective SNPs using the software GEMMA (Zhou and Stephens, 2012). The statistical model applied in this study is as follows:

(2) y = Wα + xβ + u + ε

In this expression, y denotes the phenotypic values of EWs for *n* samples, while W refers to a covariate matrix (fixed effects: top five PCs and a column of 1s) used to control population structure, α denotes a vector of corresponding effects that comprise the intercept, **x** denotes the marker genotypes, **β** refers to the effects of corresponding markers, **u** is a vector of random polygenic effects, and **ε** is a vector of random residuals. We applied the Wald statistical test to evaluate the alternative hypothesis *H*_1_: β ≠ ***0*** vs. our null hypothesis *H*_0_: β = ***0*** for each SNP, since F_wald_ = $\frac{{\hat{\beta }}^{2}}{Var\left(\hat{\beta }\right)}$.

We then performed a multivariate association analysis to directly account for the influence of genetic variants on longitudinal EWs. A mixed model in this case was also implemented using the software GEMMA.

### Statistical analyses of post-GWAS

We generated Manhattan and quantile-quantile (Q-Q) plots for EW traits using the “gap” (https://cran.r-project.org/web/packages/gap/) and “qqman” (https://cran.r-project.org/web/packages/qqman/) packages within the software R. A genomic inflation factor (λ) was also calculated using the “GenABEL” package in R (Devlin, 1999) to evaluate the extent of false positive signals.

We performed a series of linkage disequilibrium (LD) analyses to characterize causative SNPs within strong LD regions by applying the solid spine algorithm in the software Haploview version 4.2 (Barrett et al., 2005). Thus, the most significant SNP genotypes (coded 0, 1, or 2) were added as covariates to univariate and multivariate models to elucidate independent signals in step-wise conditional analyses. Annotations of candidate genes adjacent to significant SNPs were determined using the variant effect predictor (VEP) (McLaren et al., 2010) supplied by Ensembl (http://www.ensembl.org).

We calculated SNP-based heritability (${\text{h}}_{\text{snp}}^{2}$) (Lee et al., 2012b) and pairwise genetic correlations of EW traits were using a restricted maximum likelihood (REML) approach implemented in the software GCTA v1.24 (Yang et al., 2011). This enabled us to estimate the contribution of genome-wide significant SNPs to phenotypic variance based on a genetic matrix constructed from all eligible SNPs.

## Results

### Phenotypic statistics and genetic parameter estimations

Descriptive statistics for EW across the whole laying period are presented in Table 1. These data show that as the laying period increases, EW values increase faster up to 56 week of age and then subsequently increase more slowly. Results show that the highest phenotypic value for EW was attained at 80 week of age; indeed, EWs at two subsequent age points (72 week of age and 80 week of age) both exhibited higher phenotypic variation (8.13% ~ 8.74%) than at other ages with the notable exception of FEW. Pedigree-based hereditability values were also high between 28 week of age and 66 week of age, while EW56 and at other times was moderate.

**Table 1:** Descriptive EW statistics at different ages.

**Trait**	**N**	**Mean**	**SD**	**CV (%)**	**Min**	**Max**	**h^2^ (SE)**
FEW	1,052	42.44	5.06	11.92	17.00	75.00	0.31 (0.08)
EW28	1,063	57.19	3.47	6.07	46.80	68.80	0.50 (0.08)
EW36	1,063	59.35	3.28	5.53	54.00	69.70	0.53 (0.09)
EW56	1,027	60.98	4.54	7.44	35.50	77.00	0.35 (0.08)
EW66	960	60.83	4.50	7.39	42.00	78.00	0.51 (0.09)
EW72	847	60.97	5.33	8.74	42.00	86.00	0.34 (0.08)
EW80	852	62.33	5.07	8.13	39.00	84.00	0.29 (0.08)

*FEW, first egg weight; EW28, EW36, EW56, EW66, EW72, EW80, egg weight at 26, 36, 56, 66, 72, 80 weeks of age; N, number of samples; SD, standard deviation; CV, coefficient of variance; h^2^ (SE), pedigree-based heritability (standard error)*.

Estimates of SNP-based heritability as well as genetic and phenotypic correlations among EWs are presented in Table 2. Results show that estimates of SNP-based heritability were lower than those due to pedigree for all traits apart from EW80 (0.30 vs. 0.29). Genetic parameter analyses revealed that EWs at different week of age were both positively and highly interrelated, especially for two neighboring time points. In addition, FEW is poorly correlated with other traits regardless of genetic relationship or phenotypic correlation.

**Table 2:** Estimated genetic parameters for EWs across the whole laying period.

**Trait**	**FEW**	**EW28**	**EW36**	**EW56**	**EW66**	**EW72**	**EW80**
FEW	**0.31 (0.05)**	0.72 (0.09)	0.59 (0.09)	0.65 (0.11)	0.63 (0.10)	0.56 (0.14)	0.46 (0.13)
EW28	0.34	**0.35 (0.05)**	0.87 (0.05)	0.94 (0.06)	0.85 (0.07)	0.90 (0.08)	0.92 (0.08)
EW36	0.31	0.64	**0.36 (0.05)**	0.97 (0.06)	0.84 (0.06)	1.00 (0.07)	0.85 (0.08)
EW56	0.27	0.44	0.50	**0.23 (0.05)**	0.95 (0.07)	0.91 (0.11)	0.98 (0.08)
EW66	0.32	0.44	0.52	0.51	**0.32 (0.05)**	0.97 (0.07)	0.92 (0.07)
EW72	0.24	0.39	0.43	0.46	0.58	**0.22 (0.06)**	1.00 (0.11)
EW80	0.20	0.27	0.40	0.38	0.48	0.47	**0.30 (0.06)**

*Shaded diagonal values are heritability estimates (those in bold are SNP-based), while the upper triangles are genetic correlations and lower triangles are phenotypic. SE values are reported in parentheses*.

### Global genome-wide association study (GWAS)

**Figure 1:**
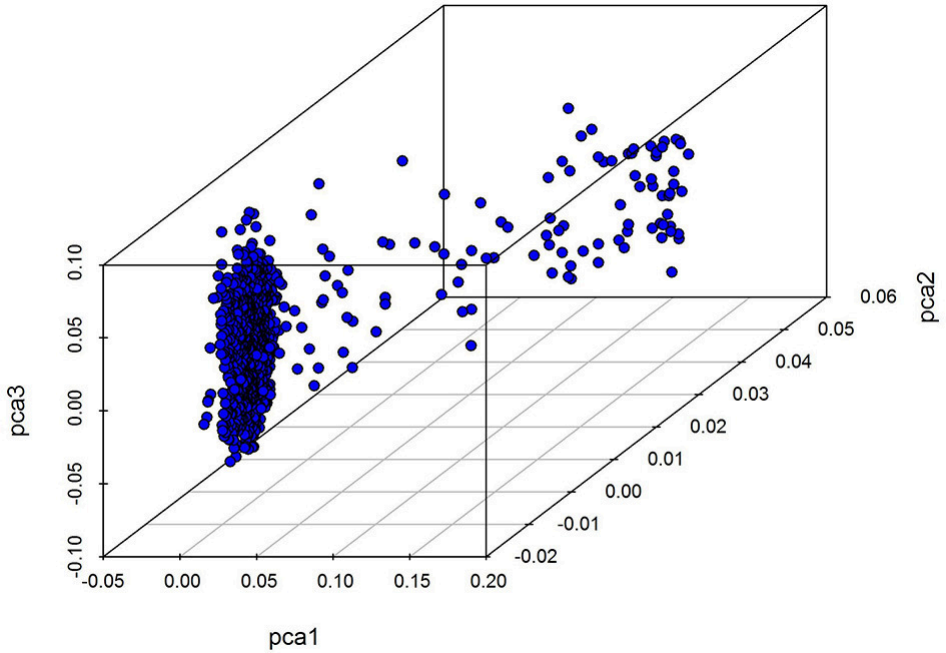
A 3D PC plot for chicken using SNP markers. The blue points denote individuals.

Our analysis of population structure is presented in Figure 1. The results of this three dimensional (3D) plot show that individuals has a slight population stratification; we therefore treated the first five principal components as covariates and included them within a GWAS linear mixed model as fixed effects in order to adjust for variations in population structure.

**Figure 2:**
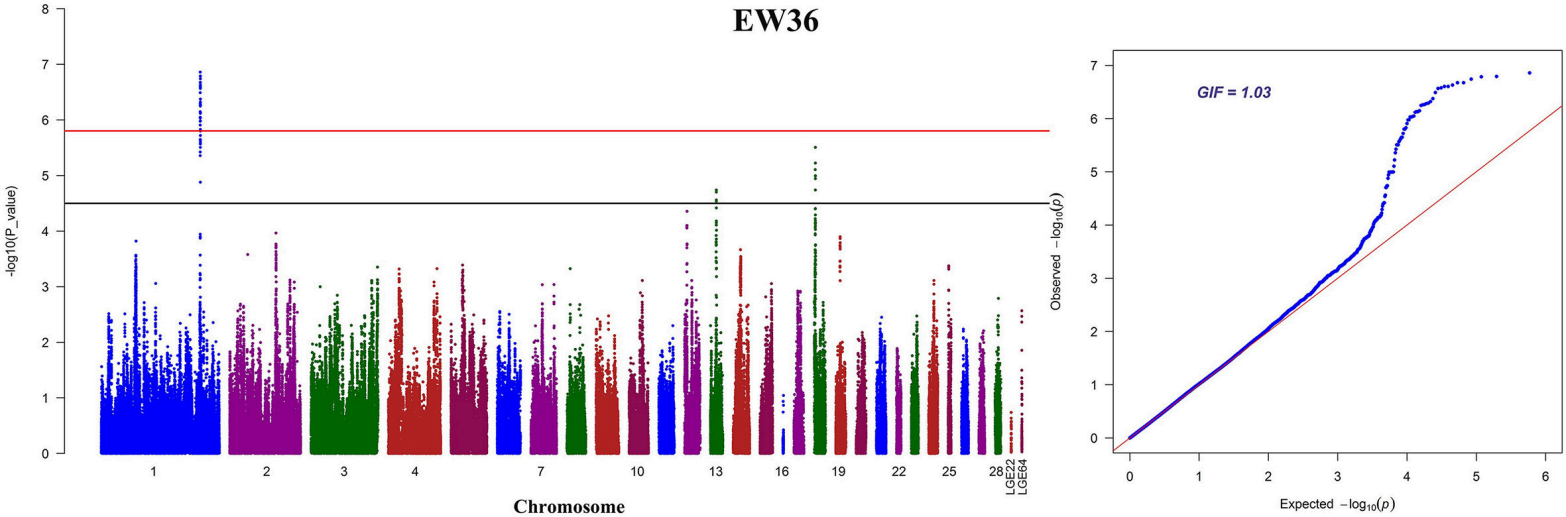
Manhattan and Q-Q plots derived from a GWAS at EW36. Each dot on this figure corresponds to a SNP within the dataset, while the horizontal red and black lines denote the genome-wide significance (1.58e-6) and suggestive significance thresholds (3.17e-5), respectively. The Manhattan plot contains –log10 observed *P*-values for genome-wide SNPs (y-axis) plotted against their corresponding position on each chromosome (x-axis), while the Q-Q plot contains expected -log10-transformed *P*-values plotted against observed –log10-transformed *P*-values. GIF denotes the genomic inflation factor.

**Figure 3:**
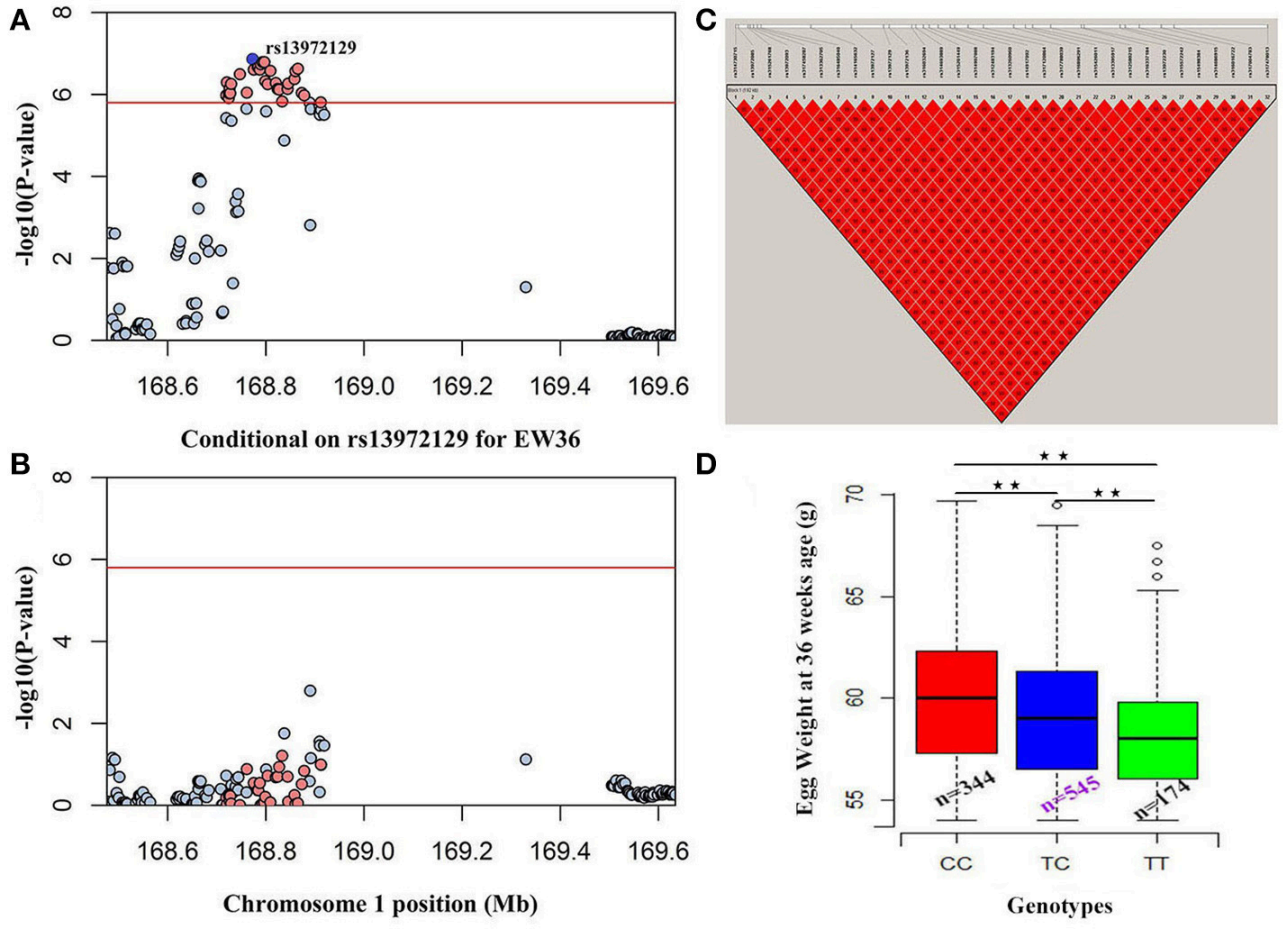
Conditional GWAS and LD analyses of SNPs in the significant region for EW36. Results **(A)** before, and **(B)** after, conditional association analyses were performed by fitting the most significant SNP rs13972129 as covariates. **(C)** LD plot of significant SNPs on GGA1. **(D)** Genotype effect plot of SNP rs13972129 (^⋆⋆^*P* < 0.01) to indicate the significance among three types (*n* = 344, *n* = 545, and *n* = 174 for CC, TC, and TT, respectively).

We performed seven separate association tests for longitudinal EW using a univariate linear mixed model. These analyses revealed a total of 65 genome-wide significant and suggestive SNPs located on GGA1 for EW36, EW56, EW66, and FEW (Table S1). Of those significant SNPs, 15 interesting loci that attain a genome-wide level of significance at 36 weeks of age are also potentially associated with EW at 28, 56, and 66 week of age in both the univariate and multivariate analyses (Figure S1). A global view of Manhattan and QQ plots for EW36 are presented in Figure 2; a high genetic correlation shows that all of these SNPs are located within a 90 Kb region that spans between 169.42 and 169.51 Mb on GGA1. We also calculated the genomic control inflation factor (λ) at EW36, a value which ideally should equal 1; the fact that this was a little higher (1.03) is indicative of slight population stratification. These results are also consistent with previous PCA results, while LD analysis reveals that all these genome-wide significant SNPs were in strong LD status (Figure 3C). Thus, in order to identify independent SNPs, a further series of stepwise conditional analyses were performed and the locus rs13972129 that is significantly associated with EW36 was fitted into the model to examine these associations. The level of significant, or suggestive loci surrounding SNP rs13972129 decreased below the genome-wide suggestive threshold when the genotype of this locus was treated as a covariate in the conditional GWAS (Figures 3A,B). We therefore suggest that the rs13972129 locus provides the most reliable signal. The substitution of variant C to T for rs13972129 led to a significant decrease of in EW phenotypic value at 36 week of age (Figure 3D).

### SNP effects and promising genes associated with EW36

Data show that the most significant SNP at EW36 (rs13972129 on GGA1) accounts for 3.66% (SE = 0.04) of phenotypic variance. Thus, annotation of the 15 interesting SNPs discussed above using the VEP tool may help us identify promising EW-associated genes. Detailed information regarding the genes identified in this study is summarized in Table 3; one candidate was deleted in lymphocytic leukemia 7 (DLEU7), a microRNA (MIR15A) was detected adjacent to significant SNPs, and we also identified several genes near to suggestively significant SNPs, including the ribonuclease H2 subunit B (RNASEH2B), a potassium channel regulator (KCNRG), and a SPRY domain containing 7 (SPRYD7).

**Table 3:** Annotation of significant SNPs associated with EW at 36 week of age.

**SNP**	**GGA^a^**	**Position^c^**	**Alt/Ref**	**MAF**	**β^b^ (SE)**	**Candidate/nearest gene**	**Location (Kb)**
rs13972129	1	169475409	T/C	0.42	0.27(0.05)	DLEU7	downstream_350.01
rs313260960	1	169499480	C/T	0.419	0.27(0.05)	DLEU7	downstream_325.94
rs312483194	1	169495186	C/T	0.421	0.27(0.05)	DLEU7	downstream_330.24
rs314907088	1	169491906	C/T	0.417	0.27(0.05)	DLEU7	downstream_333.52
rs316032694	1	169485590	G/A	0.421	0.27(0.05)	DLEU7	downstream_339.83
rs314693889	1	169487134	G/A	0.421	0.27(0.05)	DLEU7	downstream_338.29
rs317788039	1	169511502	G/A	0.419	0.27(0.05)	DLEU7	downstream_313.92
rs314165632	1	169449898	C/T	0.429	0.26(0.05)	DLEU7	downstream_376.33
rs13972085	1	169423767	A/T	0.425	0.26(0.05)	MIR15A	upstream_25.89
rs316485040	1	169431981	G/T	0.432	0.26(0.05)	MIR15A	upstream_34.10
rs317458287	1	169429248	T/C	0.433	0.26(0.05)	MIR15A	upstream_31.37
rs13972093	1	169428043	C/T	0.43	0.25(0.05)	MIR15A	upstream_30.16
rs313362705	1	169430636	C/T	0.43	0.25(0.05)	MIR15A	upstream_32.76
rs314730715	1	169422726	T/A	0.424	0.25(0.05)	MIR15A	upstream_24.85
rs315261768	1	169427284	G/A	0.424	0.25(0.05)	MIR15A	upstream_29.40

a Chicken chromosome; Alt/Ref, alternative allele/reference allele;

b *Estimated allelic substitution effect per copy of the effect allele (EA) based on an inverse-normal transformed scale under an additive model, expressed in SD unit/allele*.

c *Gallus_gallus-5.0*.

### GWAS for FEW and EW56

**Figure 4:**
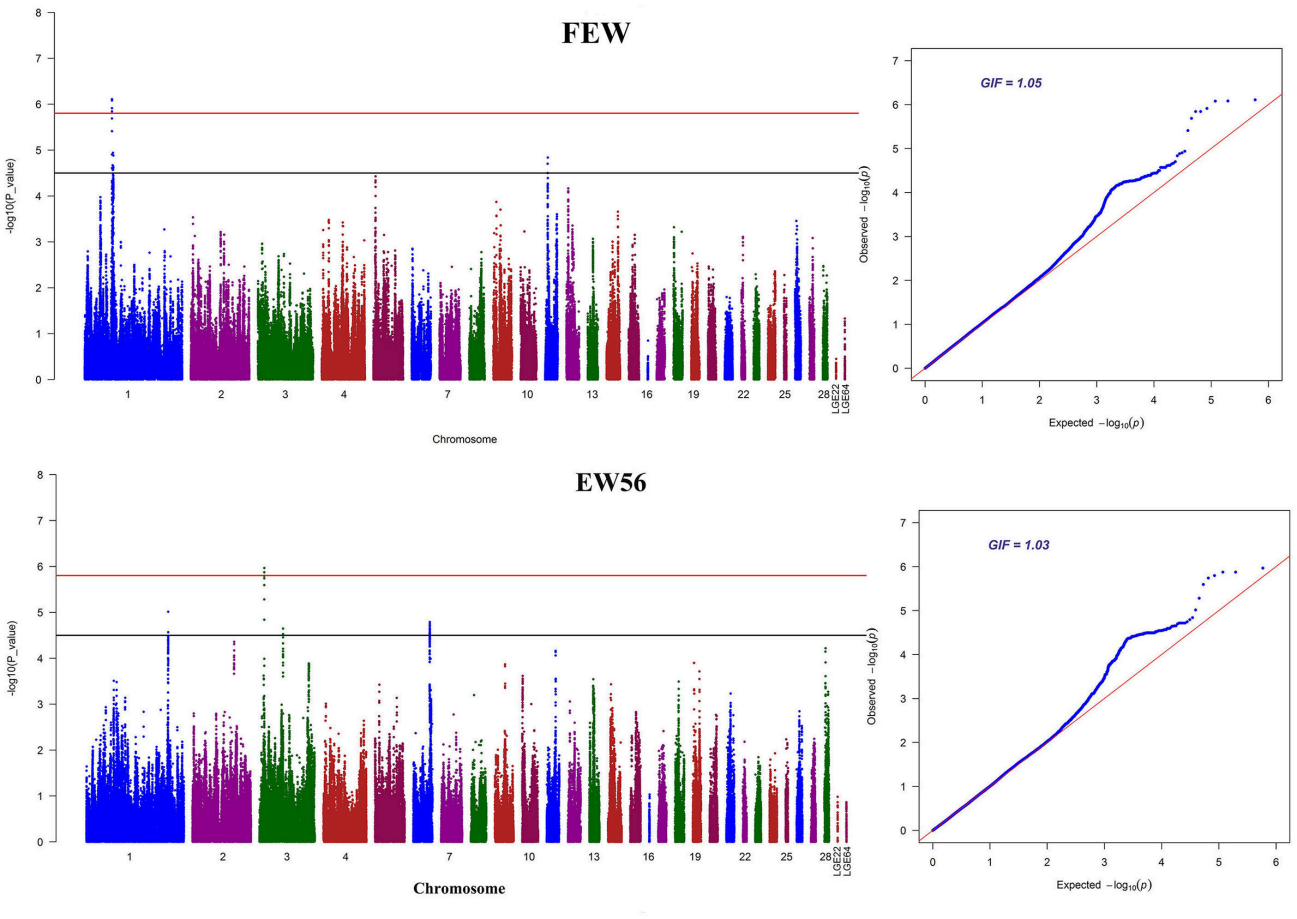
GWAS Manhattan and Q-Q plots for FEW and EW56. The horizontal red and black lines in this figure denote the genome-wide (1.58e-6) and genome-wide suggestive significant thresholds (3.17e-5), respectively.

**Figure 5:**
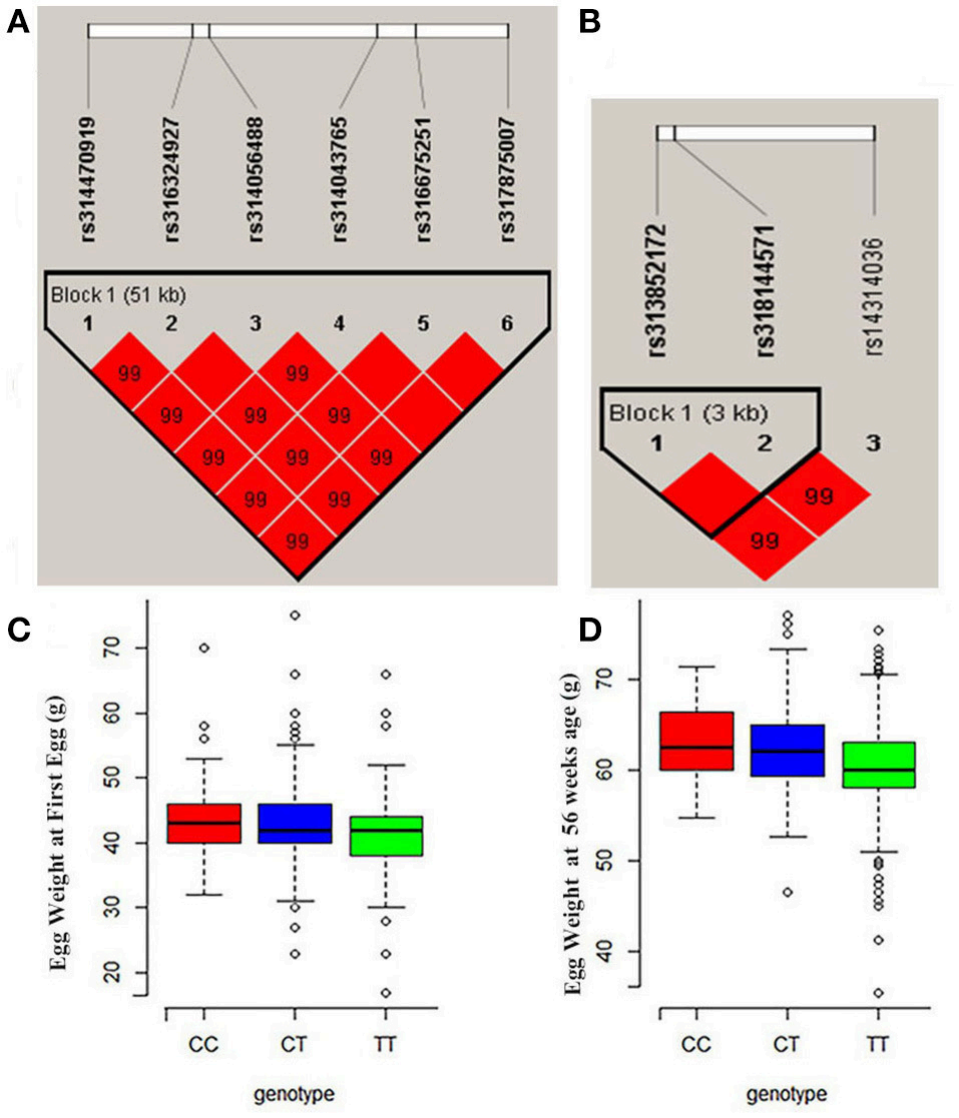
Regional association plots of significant SNPs for FEW and EW56**. (A)** LD plot for SNPs on GGA1 for FEW (the strong LD block is defined as D′ ≥ 0.8). **(B)** Significant SNPs located on GGA3 associated with EW56 were within a 3Kb block. **(C,D)** The three different genotype effects of the two leading SNPs (rs314056488 and rs14314036) for FEW and EW56, respectively.

Manhattan and QQ plots for FEW and egg weight at 56 week of age (EW56) are presented in Figure 4. Results show that nine SNPs were different with loci identified in the global genome-wide association study, including six located on GGA1 associated with FEW, while three SNPs located on GGA3 associated with EW56. All of these SNPs reached the genome-wide significance level (*P*-value 1.58e-6) in our univariate GWAS analysis and are clustered into Block 1 (51 Kb) and Block 2 (3 Kb) (Figures 5A,B). The two leading SNPs (rs314056488, rs14314036) explain 2.66% (SE = 0.04) and 2.67% (SE = 0.04) of the FEW and EW56 phenotypic variance, respectively. We therefore also compared the actual phenotypic difference among the three genotypes of these two SNPs (Figures 5C,D); these data show that SNPs with CC genotypes had higher FEW and EW56 phenotypic values than those with genotypes CT and TT. The gene information we recovered by annotating significant SNPs is presented in Table 4; it is noteworthy that half of the significant SNPs associated with FEW are located upstream of the histone acetyl-lysine reader, CECR2, while two candidate genes for the cat eye syndrome chromosome region, CECR1 and CECR5, were also identified. For EW56, the SNP rs14314036 was located in 36.86 Kb upstream of Meis homeobox 1 (MEIS1), while the other two (rs313852172, rs318144571) were located within the intron of the sprouty related EVH1 domain containing 2 (SPRED2).

**Table 4:** Genome-wide association analyses for FEW and EW56.

**Trait**	**SNP**	**GGA^a^**	**Position**	**Alt/Ref**	**MAF**	**β^b^ (SE)**	**Candidate/nearest genes**	**Location (Kb)**
FEW	rs314056488	1	61739524	C/T	0.365	0.25 (0.05)	CECR1	intron
	rs316324927	1	61737361	T/G	0.366	0.25 (0.05)	CECR1	intron
	rs317875007	1	61776091	A/G	0.365	0.25 (0.05)	CECR2	upstream_25.80
	rs314470919	1	61724564	C/A	0.366	0.24 (0.05)	CECR5	upstream_3.13
	rs314043765	1	61760180	T/C	0.368	0.24 (0.05)	CECR2	upstream_41.71
	rs316675251	1	61764799	C/T	0.368	0.24 (0.05)	CECR2	upstream_37.09
EW56	rs14314036	3	10116322	C/T	0.164	0.32 (0.06)	MEIS1	upstream_36.86
	rs313852172	3	10067518	A/G	0.167	0.32 (0.06)	SPRED2	intron
	rs318144571	3	10071463	C/T	0.167	0.32 (0.06)	SPRED2	intron

*Abbreviations and symbols as in Table 3*.

## Discussion

Although a number of previous studies have attempted to reveal the genetic determinants of EW in chicken, most have focused on just a single or pair of time points. This means that little information is available regarding longitudinal EWs over the whole of the laying period (Wright et al., 2006; Liu et al., 2011). In earlier work, Yi et al. (2015) performed a GWAS series to detect the genetic architecture of EWs at different ages within a F2 crossed population using the Affymetrix 600 K SNP chip (Yi et al., 2015). An interesting region that harboring *NCAPG* has not been identified in our study. We suggest that this region may be associated with egg weights of White Leghorn after we searched QTLs that affecting EW on GGA4 in chicken QTL database (https://www.animalgenome.org/cgi-bin/QTLdb/GG/ontrait?trait_ID=2292). In addition, these workers, however, were mainly concerned with the phenotypes of laying hens younger than 60 weeks of age, a point when egg production yield remains high (Figure S2). As the aim of this study was to identify the key genes that affect EWs at seven time points (FEW, EW28, EW36, EW56, EW66, EW72, and EW80), we are the first to perform EW GWAS throughout the extended laying cycle employing a chicken 600 K high density SNP array.

Because a population line of purebred brown egg-type chickens was used in this study rather than a cross between two, or more, distant populations which have larger phenotypic variation than the pure line, our power to detect EW QTLs was diminished (Zhang et al., 2017). Nevertheless, this shortcoming was compensated for by the numerous genotypes and phenotypes of individuals and our use of appropriate methods (Alipanah et al., 2013). Descriptive phenotype statistics show that EW increases in concert with the age of a hen, reaching the highest value at 80 week of age. We are therefore able to predict that if the egg laying cycle was extended to 100 weeks, EW would continue to increase. It remains a challenge, however, to maintain the stable weight of egg across the whole laying period. The SNP-based heritability estimates for EW presented in this paper are lower than those reported for an F2 crossed population, while genetic correlations are the same (Yi et al., 2014); the presence of a strong genetic correlation between the seven EW traits discussed in this study assumes that they all encapsulate similar components of genetic variations (Lu et al., 2012; Fu et al., 2015) and our pedigree-based heritability estimates were larger than their SNP-based counterparts. This difference may be the result of “missing heritability” (Manolio et al., 2009) as eligible SNPs within the 600 K chip do not encapsulate the whole of chicken genomic variation.

We performed separate GWAS series for EWs at seven time points in this study. Results show that a 90 Kb genomic region on GGA1 harbored 15 genome-wide significant SNPs and was associated with EW at 36 week of age. These SNPs were also determined to be related to EW at 28, 56, and 66 week of age but were not significant (Figure S1). Multivariate tests also show that these significant SNPs are responsible for all these phenotypes, although no significant hit was identified at EW72 and EW80. This may be caused by some missing phenotypes or hens may don't lay eggs anymore at 72 and 80 weeks age (Lee et al., 2012a; Zhou and Stephens, 2012). Conditional GWAS and LD analyses at EW36 revealed SNPs that are closely linked together within this significant genomic region; the annotation of significant SNPs shows that one candidate gene, DLEU7, and a promising microRNA (MIR15A) around this region are both also associated with EW36. Previous research of GWAS report that the DLEU7 gene was related to ovary weight in chicken (Sun et al., 2015). In addition, studies on humans have also shown that DLEU7 is associated with height during body growth and other developmental processes, although specific physiological mechanisms remain unclear (Weedon et al., 2008; Kang et al., 2010; Fatemifar et al., 2013). We therefore suggest that the DLEU7 gene should be considered as a candidate associated with EW and subjected to further functional validation in chicken. In addition, one other microRNA, MIR15A, is also known to be an important independent regulatory molecule involved in the control of cell proliferation and apoptosis, cardiovascular and autoimmune diseases, and the synthesis of insulin (Andersen et al., 2010; Sun et al., 2011; Yuan et al., 2012; Spinetti et al., 2013). During chick embryonic development, the inhibition of MIR15A or the activation of HIF-1 or Bcl-2 can prevent hypoxia-induced lung damage and reduce the number of chick embryonic deaths (Hao et al., 2014). Yuan et al. (2017) noted that MIR15A can also control the feed conversion ratio in laying chickens and exerts an influence on a number of target genes, including forkhead box O1 (FOXO1) which is also involved in the insulin-signaling pathway (Yuan et al., 2017). This pathway stimulates protein synthesis and cell growth via mTOR signal activation (Kim et al., 2002); we therefore suggest that MIR15A has an indirect effect on longitudinal EW by affecting the deposition of egg white and yolk.

It is noteworthy that just six and three hits, respectively, were detected for FEW and EW56 at a genome-wide level of significance. Indeed, in the first case (FEW), just 50% of significant SNPs were located upstream of the CECR2 gene. Previous research has shown that uncovered that CECR2 is expressed during chicken embryonic development and exerts and influence on both somites and neurons (Footz et al., 2002; Banting et al., 2005; Chen et al., 2010). Limited information is presently available regarding the CECR1 and CECR5 genes in chicken. For EW56, SNP rs14314036 on chromosome 3 (GGA3) is located adjacent to a QTL previously identified in an F2 population (Yi et al., 2015), and that two additional and interesting genes related to EW are also present at 56 week of age. The MEIS homeobox genes (MEIS1 and MEIS2) belong to the three-amino-acid loop extension (TALE) superfamily which has been subdivided into IRO, MKX, TGF1, PBC, and MEIS classes (Sánchez-Guardado et al., 2011a). These genes are important because they regulate various developmental processes by promoting cell proliferation, repressing differentiation, and preventing cell fate specification via a number of signaling pathways (Bessa et al., 2008; Heine et al., 2008; Sánchez-Guardado et al., 2011b). In chicken, MEIS1 plays a crucial role during early embryogenesis and organogenesis, while another gene, the sprouty related EVH1 domain containing 2 (SPRED2), is a member of the SPRED gene family (SPRED1, SPRED2, and SPRED3) (Bundschu et al., 2007). SPRED2 is widely expressed in adult tissues, including the liver and brain (Kato et al., 2003; Ma et al., 2011), and may act to modulate cellular proliferation and migration (Ma et al., 2011). We therefore speculate that the genes CECR2, MEIS1, and SPRED2 may act to influence chicken EW during embryogenesis and organogenesis.

In conclusion, the GWAS presented in this study demonstrates that EW is highly heritable and shares similar genetic determinants at different week of ages. Two promising genes, DLEU7 and MIR15A, that may influence EW in a pleiotropic manner were also mapped to within a narrow region of significance, while three additional candidates (CECR2, MEIS1, and SPRED2) identified by annotating nine significant SNPs can be considered as candidates related to FEW and EW56. Findings in our research could provide valuable breeding theory for the future production of ideal egg size in the context of marker-assisted breeding selection.

## Data accessibility

The genotype and phenotype data of the samples used in this study are available from the FigShare Repository: https://figshare.com/articles/Genome-wide_Association_Analysis_of_Age-Dependent_Egg_Weights_in_Chickens/5844420.

## Author contributions

NY: Conceived the study and designed the project; ZL: Performed the genetic and bioinformatics analyses; ZL, YY, GL, GW, and AL: All contributed by collecting samples and measuring phenotypic data; ZL: Wrote the manuscript, which was then revised by NY and CS. All authors read and approved the final draft.

### Conflict of interest statement

The authors declare that the research was conducted in the absence of any commercial or financial relationships that could be construed as a potential conflict of interest. The reviewer XSS and handling Editor declared their shared affiliation.
