# Supplementary material for: Genome-Wide Association Analysis of Age-Dependent Egg Weights in Chickens
Source: Front Genet. 2018 Apr 26;9:128. doi: 10.3389/fgene.2018.00128 (PMC5932955; doi:10.3389/fgene.2018.00128)

Supplementary Figure S1

A. Manhattan plot and Q-Q plot of genome-wide association study for EW26.

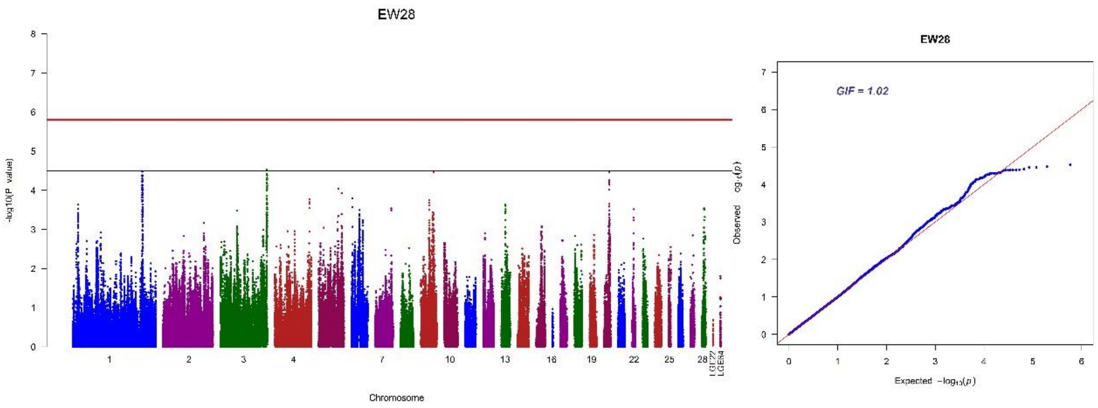

B. Manhattan plot and Q-Q plot of genome-wide association study for EW66.

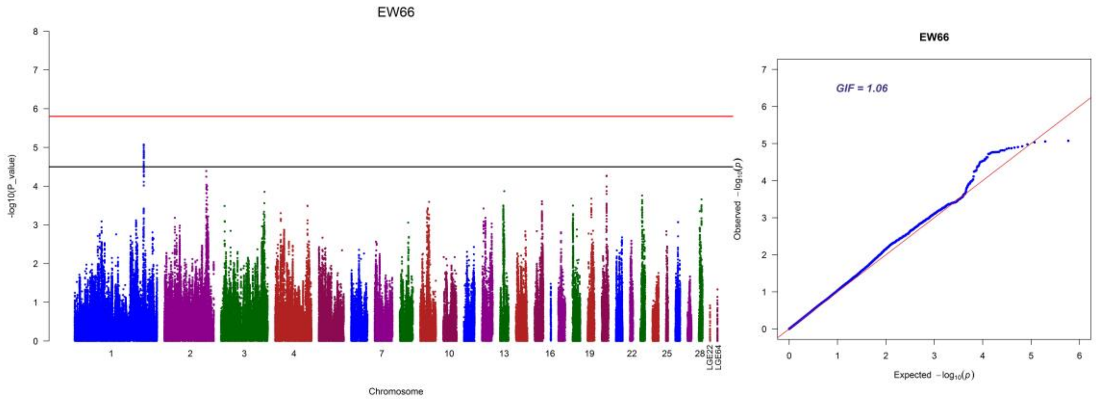

C. Manhattan plot and Q-Q plot of genome-wide association study for EW72.

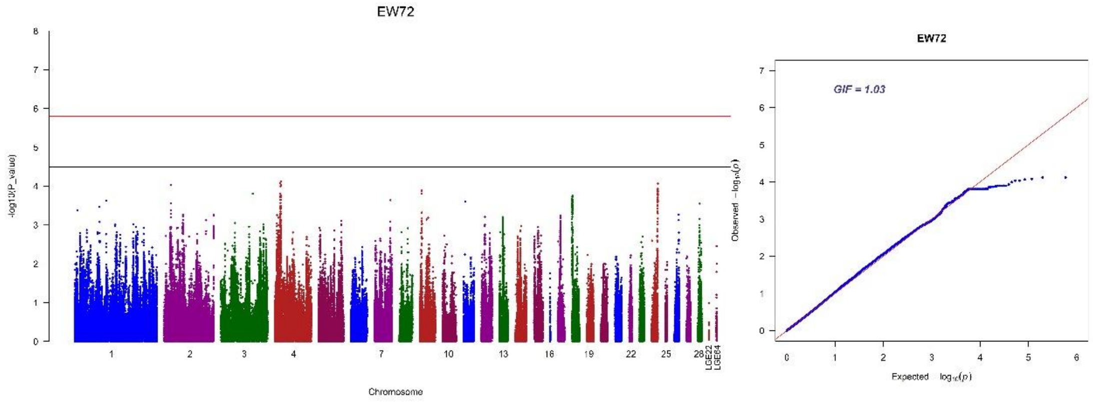

D. Manhattan plot and Q-Q plot of genome-wide association study for EW80.

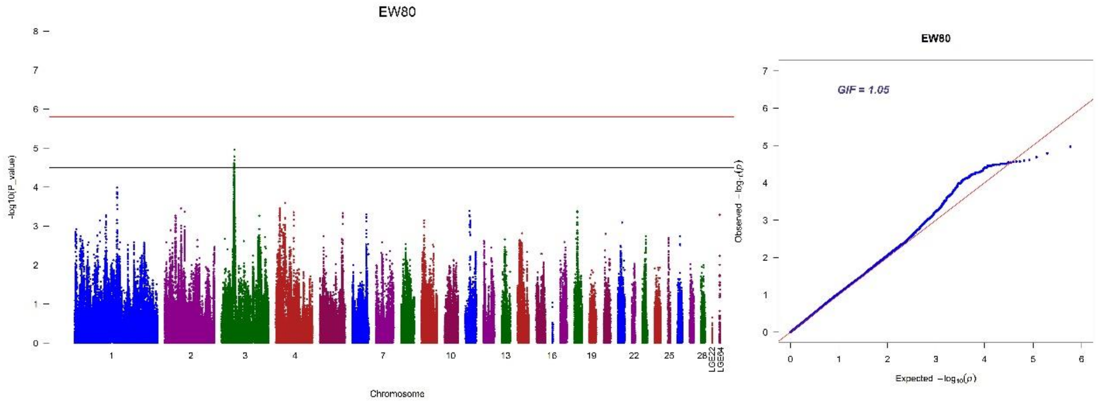

E. Manhattan plot and Q-Q plot of genome-wide association study for Egg weight at muti-ages.

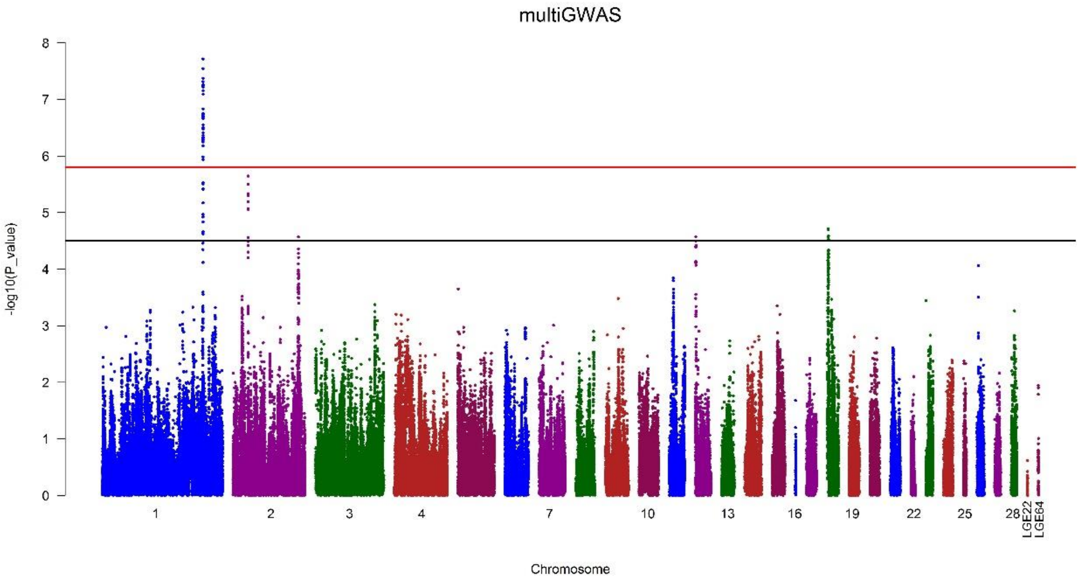

Supplement: Supplementary file 1 [file Image1.pdf]
