# Supplementary figures and images for: Genome-Wide Association Analysis of Age-Dependent Egg Weights in Chickens
Source: Front Genet. 2018 Apr 26;9:128. doi: 10.3389/fgene.2018.00128 (PMC5932955; doi:10.3389/fgene.2018.00128)

### Supplementary Figure S2

The rate of egg production in the whole laying period.

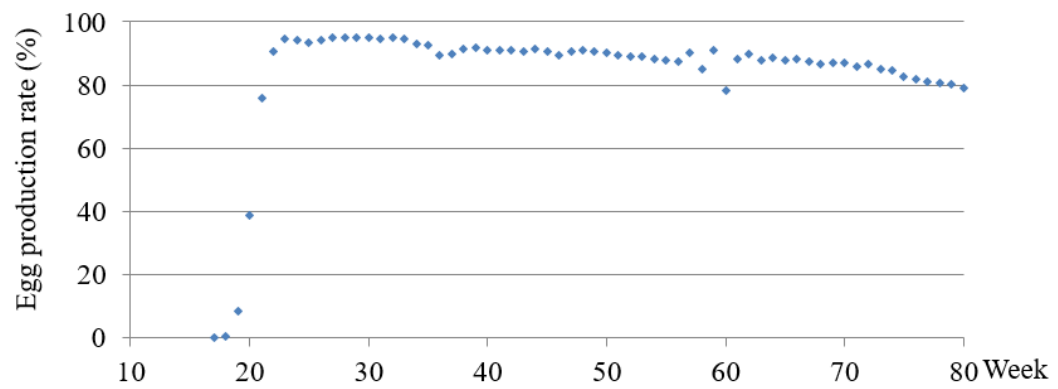

Supplement: Supplementary file 2 [file Image2.pdf]
